# Does motivation matter? – The relationship between teachers’ self-efficacy and enthusiasm and students’ performance

**DOI:** 10.1371/journal.pone.0207252

**Published:** 2018-11-21

**Authors:** Daniela Mahler, Jörg Großschedl, Ute Harms

**Affiliations:** 1 Leibniz Institute for Science and Mathematics Education (IPN), Department of Biology Education, Kiel University, Kiel, Germany; 2 Institute for Biology Education, Department of Science and Mathematics Education, University of Cologne, Cologne, Germany; Waseda University, JAPAN

## Abstract

Knowledge and motivation of a teacher are two unchallenged, essential characteristics for successful education. Whilst the relevance of teachers’ professional knowledge for successful students’ learning has been studied in a sophisticated manner for years, the meaning of teachers’ motivational orientations for students’ performance still lacks a differentiated consideration. This construct is conceptualized by three domains: (1) self-efficacy, (2) subject-specific enthusiasm, and (3) enthusiasm for teaching the subject. Motivational orientations overall have shown to be relevant predictors of students’ learning. However, there are several dimensions of motivation and their relative importance remains unclear. Our study goes beyond the available findings by considering in detail each of the three domains’ relations to students’ performance. Thus, we aim to further contribute to the clarification of the predictors of students’ performance in school teaching. For this purpose, we conducted a study with 48 biology teachers and their 1036 students. To assess the three domains of teachers’ motivational orientations, we applied paper and pencil tests. Concept maps and paper and pencil tests were used to measure students’ performance. By specifying multilevel structural equation models, we examined the relationship between the domains of teachers’ motivational orientations and the performance of the students. Our results reveal no relationship between teachers’ self-efficacy and students’ performance, but a significant positive relationship between the latter and teachers’ subject-specific enthusiasm. Moreover, our results show a positive trend in the relationship between enthusiasm for teaching the subject and students’ performance. The results provide a differentiated picture about the importance of motivational orientations for the characterisation of an effective teacher. We discuss our findings in terms of possible effect mechanisms and their relevance for further research on teacher motivation and the improvement of teacher education programmes.

## Introduction

Supporting students to learn is the superordinate aim of school education. Apart from individual factors (e.g., students’ cognitive abilities), the teacher is one of the most important determinants of students’ performance (e.g., [1–4]). In fact, teachers have more impact than fiercely politically debated issues such as school budgets and class sizes [3]. This raises questions regarding the characteristics of an effective teacher. The study at hand, focuses on teachers’ motivational orientations as an important characteristic of effective teachers and their relation to students’ performance. Motivational orientations are related to the "psychological dynamics of behaviour, the maintenance of intentions, and the monitoring and regulation of occupational behaviour" ([5] p. 38). We focus on both, cognitive and affective domains of teachers’ motivational orientations, by considering teachers’ self-efficacy (e.g., [6]) as well as their enthusiasm (e.g., [7]).

Several studies have addressed the relationship between one of these domains of teachers’ motivational orientations and students’ performance (e.g., [7–10]), but no previous studies have compared the domains’ predictive power. Thus, the relative importance of the domains remains unclear. Moreover, the available studies did not connect the content of the performance tests to specific learning opportunities or lessons in which teachers’ motivational orientations become visible (e.g., [10–12]). Furthermore, few studies have considered the subject of teacher enthusiasm (subject vs. teaching the subject) [7,13,14]. The aim of this study is to contribute to the clarification of these issues by comparatively examining the relations between students’ performance and both—cognitive (self-efficacy) and affective (enthusiasm for the subject and teaching the subject)—domains of teachers’ motivational orientations [14].

### Teachers’ self-efficacy—Conceptualisation and significance for students’ performance

According to Bandura [6], in the framework of social cognitive theory, self-efficacy is an individual’s belief that he or she is capable of performing the behaviours required to produce a desired outcome. Compared to more general constructs like self-esteem, self-efficacy is related to a certain context [15]. Therefore, we consider *teachers’ self-efficacy* as a teacher’s belief in his or her ability to succeed in a teaching-related context by performing specific behaviours [15,16]. Desired outcomes within a teaching-related context concern students’ engagement and learning [16]. Further areas of interest concern: (1) the professional practice [16–19], (2) coping with job stress (see also [17,19,20]), (3) the interaction with students, parents, and colleagues [18,20] as well as (4) skill development on the job [19].

As already mentioned in the introduction, we consider teachers’ self-efficacy as the cognitive domain of teachers’ motivational orientations (cf. [14]). The consideration of information to generate an expectancy of their own efficacy represents the related cognitive process [6].

There is a broad consensus in the research literature that teachers’ self-efficacy is important for students’ performance. The relationship between teachers’ self-efficacy and students’ performance has been confirmed in language [8–11,21–23], mathematics [9–11,22,24], science [10], and social studies [10,25]. In contrast, Zinke [12] did not find a positive relationship between teachers’ self-efficacy and students’ performance (in language, arts and mathematics).

Different aspects of effective teaching are related to teachers’ self-efficacy. These aspects help to understand the significance of teachers’ self-efficacy for students’ performance. Previous studies have found that self-efficacy seems to be particularly relevant for three aspects: (1) teachers’ professional engagement [19,26], (2) effective instructional strategies [17,22–30], and (3) openness to “demanding” students [27,31].

Although several studies were able to show a positive relationship between teachers’ self-efficacy and students’ performance, research gaps become apparent. As mentioned above, the available studies in part did not connect the performance tests to the lessons in which teachers’ self-efficacy becomes evident (e.g., [10–12]). Mazlum and colleagues [23] used the learning behaviour of the students as proxy for their performance. In addition, there is a lack of research focusing on the relationship between science teachers’ self-efficacy and students’ science performance.

### Teacher enthusiasm—Conceptualisation and significance for students’ performance

Teacher enthusiasm reflects the affective part of teachers’ motivational orientations [14].

We define teacher enthusiasm as affective teacher orientation, which comprises the excitement, enjoyment and pleasure associated with both a school-subject and the activity of teaching [14]. Thus, according to this definition there are two main dimensions of teacher enthusiasm: subject-specific enthusiasm and enthusiasm for teaching (the subject) [14]. Recognition of these two dimensions facilitates a detailed examination of what teachers are enthusiastic about.

*Subject-specific enthusiasm* is defined as a topic-related affective orientation [14]. It concerns a person-object relation, in this case a teacher-subject relation. Kunter and colleagues [14] define *enthusiasm for teaching the subject* as enjoyment related to the activity of teaching a specific subject. Again, this construct concerns a person-object relation. Here, the object is the interaction with students when teaching a specific subject.

From an intuitive point of view, students, parents, and teachers themselves see enthusiasm as a crucial characteristic of effective teachers [32,33]. Moreover, there are theoretical assumptions that indicate a significance of teacher enthusiasm for students’ learning [33,34]. Firstly, teachers’ enthusiastic behaviour may increase students’ attention [35] because elements of enthusiastic behaviour (e.g., gestures and body movements [36]) reportedly catch students’ attention more effectively than other external factors, as disturbances or objects in the classroom ([37] p. 150). Secondly, students may adopt enthusiastic behaviours of their teachers, in other words, an enthusiastic teacher may serve as a role model [38]. Thirdly, in the concept *emotional contagion* [39] it is rooted that students may be “infected” by their teachers’ enthusiastic behaviour and subsequently feel enthusiastic themselves. However, it should also be noted that students’ enthusiasm is related to various student behaviours (e.g., concentration and on-task behaviour), which may increase or impair students’ performance [40].

Although intuitive and theoretical assumptions suggest a unified picture concerning the relationship between teacher enthusiasm and students’ learning, empirical evidence is mixed. It is important to consider the different operationalisations of enthusiasm on which the available studies base when reviewing them. Consequently, available studies either consider enthusiasm as an affective teacher orientation or focus on enthusiastic behaviour. Both operationalisations contribute to the understanding of the significance of teacher enthusiasm as the experience of emotions is related to the behavioural expression of these emotions [41–43].

In particular, Kunter and colleagues [14] consider enthusiasm as *affective orientation*. They [13] examined the relationships between subject-specific enthusiasm, enthusiasm for teaching the subject and classroom management/monitoring, cognitive autonomy support and social support: three facets of instructional quality, which are associated with students’ performance. The results indicate that enthusiasm for teaching is related to all three of these aspects of instructional quality, but subject-specific enthusiasm is related to just one of them (cognitive autonomy support). Kunter [7] also found evidence that both dimensions of teacher enthusiasm are positively associated with students’ mathematics performance.

Several studies refer to a *behaviour-based operationalisation* (e.g., [44]). Within these studies enthusiasm is treated as teachers’ ability to convey the positive value of the learning topic to the students [45]. The level of enthusiasm is often derived from observed behaviours, for example the teaching style [46] or gestures and facial expressions [36]. These studies often provide experimental designs in which different conditions with different levels of enthusiasm are carefully contrasted (e.g., [32,47–49]). The results of these studies are mixed. Some studies support the relevance of teacher enthusiasm (e.g., [40,49–54]), while others find no apparent or very limited effect of enthusiasm on students’ learning [32,35,47–49,55,56].

### Research questions and hypotheses

The aim of this study is to elucidate associations between teachers’ motivational orientations and students’ performance. To reach this aim, we address the following research questions:

1. How are teachers’ self-efficacy and students’ performance related?
  Teachers’ self-efficacy is reportedly an important determinant of students’ performance [8–11,21–25]. Nevertheless, further research is needed as the available studies are not specific enough concerning the content of the performance tests (e.g., [10–12]) or as they used proxy variables instead of a performance score [23]. Moreover, there is a lack of research concerning science teachers’ self-efficacy and its significance for students’ performance. Referring to the available research, we expect to find a positive relationship between this domain of teachers’ motivational orientations and students’ performance.
2. How are teachers’ subject-specific enthusiasm and students’ performance related?
3. How are teachers’ enthusiasm for teaching the subject and students’ performance related?

Teachers’ enthusiasm is intuitively considered important for students’ performance [32]. Nevertheless, further research is needed as the available empirical findings are indeed mixed [7,32,35,40,49]. The Kunter group considered the object of enthusiasm (subject, teaching the subject) [7,13,14]. Considering their results, we expect both facets of teacher enthusiasm to be positively associated with students’ performance. However, according to results presented by this group [7,13], we expect students’ performance to be related more strongly to teachers’ enthusiasm for teaching a specific subject than to teachers’ enthusiasm for the subject.

## Materials and methods

### Sample and procedure

#### Sample

48 biology teachers (75% female) with an average age of 40.9 years (*SD* = 11.0, range 24–64 years) and an average teaching experience of 11.6 years (*SD* = 10.5, range 0–42 years) participated in this study. The teachers were recruited from both academic and non-academic track schools in Hamburg and Schleswig-Holstein (northern Germany). In Germany two different types of schools are distinguished: Academic track schools that qualify their students for an academic career and non-academic track schools that qualify their students for a vocational career. Accordingly, different teacher education programmes are provided, certifying the prospective teachers for either a career at an academic track school or a non-academic track school. Of the participating schools, 54.2 and 45.8% are assigned to the academic and non-academic tracks, respectively. Similarly, 58.3 and 39.6% of the teachers were certified to teach in academic and non-academic track schools, respectively and 2.1% held a degree which is not related to teacher education (e.g., a master’s degree in biology). The teachers participated together with their 7^th^ (20.1%) or 8^th^ (79.9%) grade classes (*N* = 1036 students, 50.6% female, age: *M* = 13.5 years, *SD* = 0.73 years, range 12–16 years).

#### Procedure

The participating teachers were recruited by telephone or mail. Teachers were asked to develop and conduct a teaching unit (4 lessons) referring to the topic ‘ecosystem Wadden Sea’ with a focus on *Mytilus edulis* (Blue Mussel) and its nutrition, respiration, living conditions, development, predators, mussel fishing, and breeding (cf. [57]). To support the preparation of the lessons, the teachers received content-related information (e.g., about the nutrition of *Mytilus edulis*), desired learning goals, and materials (e.g., *Mytilus edulis*, aquaria). To increase the willingness to participate in the study, the teachers obtained incentives (blue mussels and an aquarium) which remained in their ownership for future lessons.

Before conducting the teaching unit, teachers responded to questionnaires designed to measure their self-efficacy, subject-specific enthusiasm and enthusiasm for teaching the subject. The survey took place in the teachers’ homes without time constraints. Students’ performance was measured before and after the teaching unit. As the measure involved the construction of concept maps, students received training in concept mapping for approximately half an hour.

Conducting the teaching unit has different advantages. First of all, it allows a certain level of standardisation. This is important, because we are not interested in the relationship between lesson characteristics and students’ performance, but in the relationship between teacher characteristics (i.e. motivational orientations) and students’ performance. Moreover, it ensures that teachers’ motivational orientations are reflected in the same context in which students’ performance is measured. Implementing the teaching unit further allows to control for students’ prior knowledge (knowledge assessed previous to the teaching unit). A positive side effect is that we are able to generate information about how teachers’ motivational orientations are reflected in their lesson planning activities and their teaching practice. The assumption is, that motivated teachers provide high quality lesson planning, serving as a proxy for high quality lessons. This observation will not be considered in the analysis, but we hope that it will give some extra information to further understand the assumed relationship between teachers’ motivational orientations and students’ performance. There was no direct observation of the lessons, but the teachers were asked to provide an overview of their lesson planning (using a grid), the materials they developed and applied in the lessons as well as information about specific incidents or difficulties during the lessons. For each subtopic (e.g., respiration and nutrition) teachers completed the grid providing information about planned teacher- and student activities, lesson arrangements (e.g., group work), as well as applied materials and media for each phase of the lesson. The grids are evaluated according to theoretical principles (e.g., didactical reconstruction [58]).

### Measures

In order to examine the relationships between teachers’ motivational orientations and students’ performance, we applied both teacher- and student-level measures (see Table 1 for item and scale statistics as well as S1 Measures).

**Table 1:** Measures–descriptive statistics.

Measure	No. of items	Scoring	*M*	*SD*	Cronbach’s α	Facets of knowledge	Reference
*Techer level measures*							
**Self-efficacy**	10 (closed)	Likert type (1–4)	20.51	3.30	.68	-	[61]
**Subject-specific enthusiasm**	3 (closed)	Likert type (1–4)	8.35	1.01	.78	-	[62]
**Enthusiasm for teaching the subject**	2 (closed)	Likert type (1–4)	5.27	0.93	.88	-	[62]
*Student level measures*							
**System thinking I (paper and pencil test)**	26 (22 closed and 4 open)	Dichotomous (0–1), *N* = 9; polytomous (0–2), *N* = 17	19.04 (pre); 25.50 (post)	4.71 (pre); 5,60 (post)	.71 (pre), .76(post)	(a) Structural system thinking (*N* = 18); (b) Procedural system thinking (*N* = 8)	[71]
**Verbal cognitive abilities**	20 (closed)	Dichotomous (0–1), *N* = 20; polytomous (0–2), *N* = 0	11.19	4.25	.76 (A), .81 (B)	Relationships between words	[78]
**Non-verbal cognitive abilities**	25 (closed)	Dichotomous (0–1), *N* = 25; polytomous (0–2), *N* = 0	16.78	5.90	.87 (A), .90 (B)	Figural relationships	[78]
**System thinking II (Concept map)**		*Propositions*:			-	Structural and procedural system thinking	[71]
		HIT (correct connection)	6.76 (pre), 9.00 (post)	2.97 (pre), 2.37 (post)			
		CR (correct rejection)	24.19 (pre), 25.28 (post)	1.79 (pre), 1.28 (post)			
		MISS (missing connection despite connection in reference map)	12.24 (pre), 10.00 (post)	2.97 (pre), 2.37 (post)			
		FA (connection despite missing connection in reference map)	1.81 (pre), 0.72 (post)	1.79 (pre), 1.28 (post)			

#### Teacher level—Self-efficacy

Unlike more general constructs like self-esteem, self-efficacy is related to a specific context [15]. It should be noted that the optimal level of specificity in the assessment of self-efficacy is controversial. As the level of self-efficacy varies across contexts, an instrument that measures self-efficacy in a very general manner lacks validity [14,59]. A very specific instrument (e.g., an instrument measuring self-efficacy related to teaching a very specific biological content) would only be applicable in very specific corresponding situations [60].

To measure teachers’ self-efficacy we used 10 items developed by Schwarzer and colleagues [61] inviting Likert-type responses (4 = fully applies; 3 = largely applies; 2 = does not much apply; 1 = does not apply at all). The items consider self-efficacy beliefs related to different job skills which are necessary for effective teaching: job accomplishment (e.g., *I am convinced that I am able to successfully teach all relevant subject content to even the most difficult students*.); skill development on the job (e.g., *I am convinced that*, *as time goes by*, *I will continue to become more and more capable of helping to address my students‘ needs*.); social interaction with students, parents and colleagues (e.g., *I know I can maintain a positive relationship with parents even when tensions arise*.); and coping with job stress (e.g., *Even if I am disrupted while teaching*, *I am confident that I can maintain my composure and continue to teach well*.) [61].

#### Teacher level–teacher enthusiasm

To measure this construct, we used an instrument that assesses teachers’ self-reports concerning their enthusiasm. This instrument was originally developed in the framework of the COACTIV-project [62] to assess *mathematics* teachers’ enthusiasm. As our participants were biology teachers, we reformulated the (Likert-type) items by replacing the word ‘mathematics’ with ‘biology’. Subject-specific enthusiasm was measured with three items (e.g., *I am enthusiastic about the subject biology*) and enthusiasm for teaching the subject with two items (e.g., *I teach biology with great enthusiasm*) inviting Likert-type responses (4 = fully applies; 3 = largely applies; 2 = does rather not apply; 1 = does not apply at all).

#### Teacher level—Validity check

To check the factorial validity of the measures, we examined whether the three assumed domains of teachers’ motivational orientations are also empirically separable (see also [63]). Teachers’ self-efficacy is described as a cognitive domain [14], whereas teacher enthusiasm represents the affective domain of teachers’ motivational orientations [14]. Furthermore, Kunter and colleagues [7,13,14] describe the two facets of enthusiasm (subject-specific enthusiasm and enthusiasm for teaching the subject) to be separable. Accordingly, we assume that the three above-mentioned domains represent unique domains of teachers’ motivational orientations. We compared three models using MPlus 5.21 [64]. The one-factor-model assumes self-efficacy, subject-specific enthusiasm, and enthusiasm for teaching the subject to load on one single latent factor. In the two-factor-model, the first factor covers self-efficacy, whereas both facets of teacher enthusiasm load on the second factor. In the three-factor-model, self-efficacy, subject-specific enthusiasm, and enthusiasm for teaching the subject represent single latent factors, respectively. The results reveal that the three-factor-model outperforms the other two models (see [63] for detailed results). χ^2^-statistics according to Satorra and Bentler [65] reveal that the differences between the three- and the one-factor- model (TRd = 63.89, Δdf = 3; *p* < .001) and between the three- and the two-factor-model (TRd = 15.10, Δdf = 2; *p* < .001) are significant. Latent correlations reveal a large relationship between both facets of teacher enthusiasm (*r* = .57, *p* < .001) and a medium relationship between subject-specific enthusiasm and self-efficacy (*r* = .43, *p* < .001) and enthusiasm for teaching the subject and self-efficacy (*r* = .49, *p* < .001), respectively.

#### Teacher level—Control variable

The control variable considered on the teacher level is the teachers’ (and their classes’) type of school (non-academic or academic track). Considering the track has a dual function: As already mentioned (see “Sample”), two types of schools are distinguished in Germany. Academic track schools certify their students for an academic career, whereas non-academic track students prepare their students for a vocational career. Different teacher education programmes exist to prepare prospective teachers for a career at one of the respective types of schools. Accordingly, there are differences related to the performance of the students [66] *and* to different teacher characteristics like performance [67–70] and motivation [70]. These differences are considered in the model when adding the track as a control variable.

#### Student level—Conceptualisation and measurement of students’ performance

**Fig 1:**
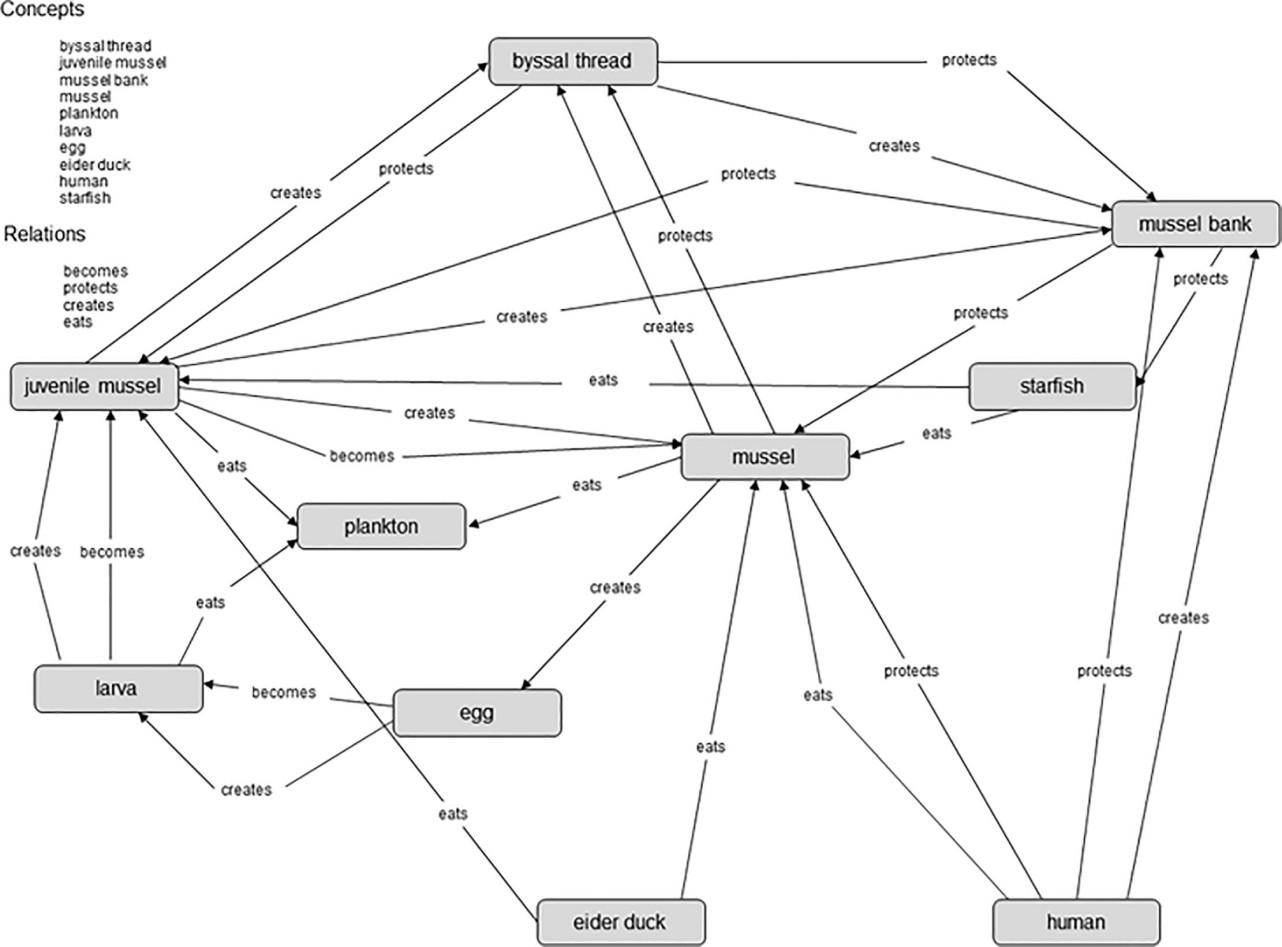
Reference map related to nutrition, living, and reproduction of *Mytilus edulis*.

Students’ performance is conceptualised to be very closely related to the content of the teaching unit. As ‘performance’ is a very vague construct, we considered a specific aspect of students’ performance: thinking about complex systems in biological contexts (*system thinking*). System thinking represents a crucial ability in biology education. The Wadden Sea ecosystem was selected as an exemplary complex system, partly because it is widely used for such pedagogical purposes in Northern Germany. To measure students’ performance in this respect we used a paper-and-pencil test as well as concept maps. The paper-and-pencil test was validated in a prior study [71] and includes two subscales. One subscale is intended to measure *structural system thinking*, covering the ability to identify, connect, and organise the elements of a system in a reference framework [71,72]. The other one is intended to measure *procedural system thinking*. This covers the following abilities: to differentiate between the properties of a system and properties of its elements; to identify dynamic relationships and to understand and predict consequences of changes within a system; and to understand and evaluate effects and reactions within a system [71,72]. Concept maps are diagrams displaying concepts (key terms of a topic) linked by arrows with appropriate labels (e.g., in this context *Eat* or *Breed*). We examined the most valid way of applying concept maps in a prior study [71], in which a paper-and-pencil design and a computer based-design were compared. We also compared the utility of highly-directed and non-directed computer-based designs (in which the software does and does not provide concept and relation options) in the previous study. The results indicated that a highly-directed computer-based design is the most valid in this context. Hence, we used such concept maps constructed with MaNet software [73] for the present study. The software provides 10 concepts (e.g., starfish and blue mussel, Fig 1) and four relating words (e.g., creates, Fig 1). The students were trained to use this software in advance.

Using a tool provided by MaNet [73] we analysed students’ concept maps by comparing each of them to a reference map (Fig 1) developed with the help of biology and biology education experts, following procedures also described by Mahler and colleagues [74]. This tool generates a weighted correspondence coefficient (C_w_) that indicates the similarity between a reference map and another map [73], based on the presence of connections between pairs of concepts (yes or no), selected relations (right or wrong), and the correctness of relations’ directions, e.g., starfish eats mussel (right) or mussel eats starfish (wrong) [73]. The weighting of the coefficient adjusts for the variation in significance of relations in maps, which declines with an increase in the number of relations [73]. This weighted coefficient was used in the following analysis.

A problem with the applied software is that it is only possible to define *one* correct relationship between a given pair of linked concepts. As our set of concepts and relating words allow different correct answers, we had to recode some of the concept maps to ensure test fairness. To evaluate concept maps with alternative but correctly related words, we defined an alternative reference map that included the dummy “correct” instead of a relating word. We then recoded the student maps by changing alternative but correctly related words using the dummy “correct” as well. This enabled us to consider different correct alternatives.

The paper and pencil test as well as the concept maps were applied in an analogous manner, both previous to the teaching unit (serving as pre-test) as well as subsequently after the teaching unit (serving as post-test).

#### Student level—Control variables

In addition to the control variable on teacher level, we considered further control variables on student level. Cognitive abilities are important determinants of students’ performance [75–77]. We used the KFT 4–12 R instrument [78] to measure students’ cognitive abilities in two dimensions: verbal abilities (relationships between words) and non-verbal abilities (figural relationships). The KFT 4–12 R is based on the Cognitive Abilities Test developed by Thorndike and Hagen [79] and has been widely used in Germany, for instance, in the national supplements of the PISA study 2000 and 2003 [80]. To avoid copying, two versions of the KFT 4-12R were given to the students.

As performance predicts future performance (e.g., [81]), we further considered the results of the pre-test.

Table 1 provides information about the measures.

### Multilevel analysis

All calculations were conducted with Mplus [64].

#### Multilevel data structure

Since teachers participated together with their classes, the acquired data (S1 Dataset) had a hierarchical structure, i.e. data pertaining to one level (individual student) were clustered into another (class) [82]. In such structured datasets observations are not independent and to avoid errors in the estimation of effects and standard errors it is highly important to account for the hierarchical data structure [82]. We specified unconditional models to decompose the variance in students’ performance into the proportions lying within and among the classes. As we used two types of instruments to measure students’ performance (paper-and-pencil-tests and concept maps), scores obtained were used as latent indicators to model students’ performance. Accordingly, we constructed separate unconditional models based on the structural system thinking, procedural system thinking and concept map (correspondence coefficient) scores. The results indicate that between-class variance accounts for 27.3, 22.7 and 23.9% of the total variance in these scores. Accordingly, we specified models that account for a multilevel structure.

#### Doubly-latent models

To investigate if teachers’ motivational orientations are predictive for students’ performance, we specified doubly-latent models [83]. The dependent variable is treated as a latent trait on both individual and class levels. Using doubly-latent models has different advantages. First of all, they consider the multilevel structure of the data. Moreover, as they integrate a structural equation model and a multilevel model, they allow to compute models with latent variables. A further advantage of such models is that they control for both, measurement error (arising from sampling on both levels) and sampling error (arising from sampling individuals in the aggregation of individual to class level) [83].

**Fig 2:**
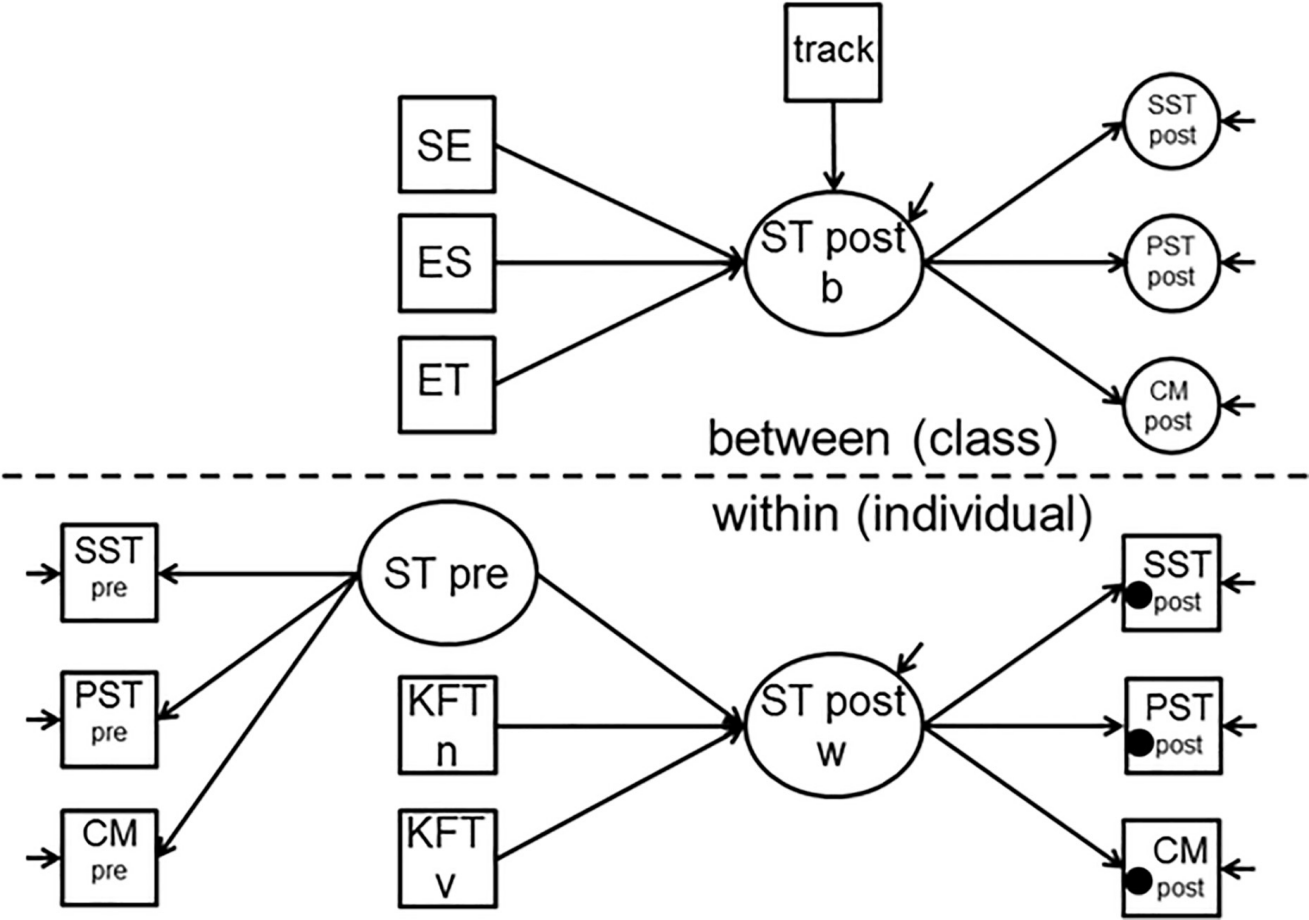
Doubly-Latent model. SE = self-efficacy, ES = subject-specific enthusiasm, ET = enthusiasm for teaching, ST = system thinking, SST = structural system thinking, PST = procedural system thinking, CM = concept mapping performance, KFT n = non-verbal cognitive abilities, KFT v = verbal cognitive abilities, pre = pre-teaching unit test, post = post-teaching unit test, ● = Intercept (DV on indicators) is modelled as a random effect, varying between classes.

The *dependent variable* here is the students’ performance (post-teaching unit) modelled with structural system thinking, procedural system thinking and concept map scores (correspondence coefficient) as indicators. As required for a doubly-latent model, the dependent variable is considered as a latent trait on both individual level and class level. The indicators of the dependent variable appear as latent variables on the class level (Fig 1). More detailed, the variance between classes within these indicators (intercept modelled as random effect) is added as a latent variable at the class level. To examine the relationship between the teachers’ motivational orientations and students’ performance, according to our research questions teachers’ self-efficacy, subject-related enthusiasm, and enthusiasm for teaching the subject are considered as *independent variables* within the models. We further included the *control variables* mentioned above (Fig 2).

We specified five models. Model 1 considers only the control variables on both levels. The first research question concerns the relationship between teachers’ self-efficacy and students’ performance. Accordingly, model 2 considers, in addition to the control variables, teachers’ self-efficacy as independent variable. The second and third research questions refer to the relationship between the two facets of teacher enthusiasm and students’ performance. Consequently, model 3 and 4 consider the control variables and subject-specific enthusiasm (model 3) and enthusiasm for teaching the subject (model 4), respectively. Finally, the control variables and all three domains of teachers’ motivational orientations are considered in Model 5 (illustrated in Fig 2 as an example of the models).

#### Missing values

Our dataset includes missing values on different variables (concept mapping performance pre: 11.7%, concept mapping performance post: 14.6%, cognitive abilities verbal: 0.19%, cognitive abilities non-verbal: 0.78%, self-efficacy: 5.0%, enthusiasm for teaching biology: 4.2%). Deleting participants with missing values reduces the sample size and weakens the statistical power [84]. In order to avoid this, we used *multiple imputation* to estimate the missing values. The underlying principle is that a certain number of datasets are generated (here: *N* = 10) in which plausible values substitute the missing values. The consideration of more than one dataset in this procedure (*multiple* imputation) accounts for uncertainty in the imputation of plausible values. The following analysis is computed with each of these ten data sets. The estimates are combined to generate a single result.

### Ethics statement

All participants participated voluntarily and gave their consent for inclusion prior to the beginning of the study. Consent forms of the parents for the participation of their children has been obtained. The purpose of the study (collection and analyzation of data for a Ph.D. thesis) was explained in advance. Contact information was collected only for the purpose of acquisition of participants and the distribution of materials. This information has been linked at no time to the other data of the study. The study was conducted in accordance with the Declaration of Helsinki. No approval by the Ethics Committee of Kiel University was necessary because the testing was anonymous and proceeded in familiar settings (schools). In addition, the study had been authorized by the “Ministerium für Bildung und Kultur des Landes Schleswig-Holstein” considering §§ 32 and 62 section 2 No. 4 of the school law of Schleswig-Holstein (Ministry of education and culture Schleswig-Holstein, Germany).

## Results

The results of model 1 reveal that students’ performance in the pre-test as well as their verbal and non-verbal cognitive abilities are significantly positively related to students’ performance in the post-test. Accordingly, we considered these control variables in the further models. The results of model 2 reveal no relationship between teachers’ self-efficacy and students’ performance. Accordingly, the consideration of teachers’ self-efficacy as independent variable does not contribute to the explanation of variance. Model 3 considers teachers’ subject-specific enthusiasm as independent variable and shows a significant positive relationship between subject-specific enthusiasm and students’ performance. Including subject-specific enthusiasm in the model does markedly contribute to the explanation of variance (ΔR^2^ = 20%). In model 4 teachers’ enthusiasm for teaching the subject was added as independent variable. Although we found no significant positive relationship, the contribution to the explanation of variance when compared to model 1 (ΔR^2^ = 8%) indicates at least a positive trend. To ensure that model 3 and 4 significantly differ from each other (to make assumptions concerning the different roles of the two facets of teacher enthusiasm), we applied a χ2 –difference test. The results reveal that these two models significantly differ from each other (χ^2^[2] = 46.56, *p*>.001). The same relationship-patterns remained in Model 5, which considers all three domains of teachers’ motivational orientations. See Table 2 for detailed results.

**Table 2:** Results of the multilevel analysis (standardised regression coefficients; standard errors in parenthesis).

Parameter	Model 1	Model 2	Model 3	Model 4	Model 5
*Individual level*					
**Pre-test**	.83*** (.05)	.83*** (.05)	.83*** (.05)	.83*** (.05)	.83*** (.05)
**KFT verbal**	.14*** (.03)	.14*** (.03)	.14*** (.03)	.14*** (.03)	.14*** (.03)
**KFT nonverbal**	.19*** (.04)	.19*** (.04)	.19*** (.04)	.19*** (.04)	.19*** (.04)
***R***^**2**^	.77	.77	.77	.77	.77
*Class level*					
**Self-efficacy**		-.02 (25)			-.25 (.28)
**Subject-specific enthusiasm**			.41** (.14)		.41*(.20)
**Enthusiasm for teaching**				.28 (.18)	.16 (.29)
**Track**	.53*** (.15)	.53*** (.15)	.56*** (.14)	.53*** (.15)	.56*** (.13)
***R***^**2**^	.28	.28	.48	.36	.53

*p < .05.

**p < .01.

***p < .001.

## Discussion

Contrary to our hypothesis and previous findings [8–11,21,22,24,25], we did not find a significant relationship between teachers’ self-efficacy and students’ performance. We assume that the specificity of our instrument causes problems. The instrument probes teachers’ self-reported efficacy with respect to important tasks in their professional lives, but not to tasks related to a specific subject (in our case biology). In contrast, the instrument we developed to measure students’ performance focuses strongly on biological topics. We surmise that the participating teachers did not necessarily think about teaching biology in general, or of the specific biological content in particular when they completed the questionnaire. Moreover, some of the items concern areas that are not directly related to teaching (e.g., interaction with parents). The instruments developed by Riggs and Enochs [85] (elementary science teachers) and Rabe and colleagues [59] (physics teachers) could give a good example for the development of an instrument that fits our needs.

Regarding the two considered dimensions of teacher enthusiasm, our results indicate that students’ performance was positively related to the teachers’ subject-specific enthusiasm. Studies with experimental designs (e.g., [32]) give hints that teachers’ motivational orientations predict students’ performance. Following this assumption, we carefully conclude that subject-specific enthusiasm could be significant for students’ performance and should thus be fostered in pre- and in-service teacher education. We know from a prior study that pre-service and in-service teacher education programs provide opportunities to develop self-efficacy and enthusiasm, e.g., the attendance in professional development courses or the conduction of self-study [63]. Thus, we assume that opportunities to develop motivational orientations in teacher education programmes could be increased by providing additional courses with an appropriate focus. Carefully following the assumption that teachers’ subject-specific enthusiasm is directly related to students’ performance, it would be fruitful to consider recent topics (e.g., epigenetics) in teacher education. Underlining the relevance of a certain content for teaching could further help to increase preservice teachers’ subject-specific enthusiasm.

On the contrary, the results reveal no relationship between enthusiasm for teaching the subject and students’ performance. It is especially interesting to compare our results to those presented by Kunter and colleagues [13] and Kunter [7], because they also conceptualised enthusiasm as affective teacher orientation and differentiated between subject-specific enthusiasm and enthusiasm for teaching a specific subject. Their findings for mathematics education—in contrast to our results but in accordance with our hypothesis–state that enthusiasm for teaching is particularly associated with students’ performance. As Kunter and colleagues are the only other group who has distinguished these facets, further research is needed to clarify the inconsistency of the results. Theoretical approaches have been developed in an attempt to describe how teacher enthusiasm is associated with students’ performance in detail, e.g., through emotional contagion [38], students’ on task-behaviour [32] or teachers’ enthusiastic behaviour increasing students’ attention [49]. These approaches treat teacher enthusiasm as instructional behaviour, rather than as affective teacher orientation as we do in the study at hand. Nevertheless, we think that these theoretical approaches are also helpful for addressing the mechanisms behind the relationship we detected between teachers’ enthusiasm and students’ performance.

To gain a deeper understanding of how teachers’ motivational orientations influence students’ performance, the possibility of a reverse effect should also be considered. By doing so, the available inconsistent results of experimental studies are also taken into account. For example, teachers’ enthusiasm may be enhanced by teaching high-achieving students [37,86]. This may also hold for teachers’ self-efficacy. Bandura [6] has shown that there are multiple sources of self-efficacy and describes mastery experiences (which occur when an individual has success in performing a specific task) as important sources for teachers’ self-efficacy. Thus, it seems plausible that teachers’ self-efficacy may be influenced by the achievements of their students.

### Limitations

Concerns are related to the instrument we used to measure teachers’ self-efficacy. As mentioned above, the instrument is broad and not specific to biology teaching. An instrument that is more closely linked to teaching biology and the biological content could have provided deeper insights into the relationship between teachers’ self-efficacy and students’ performance.

As we applied a cross-sectional design in our study, a further limitation is that we cannot draw causal conclusions concerning the relationship between teachers’ motivational orientations and students’ performance. The clarification of causality would benefit from an experiment.

As mentioned in the method section we asked the teachers to provide information about the lessons they conducted during the teaching unit to gather an insight into their planning and teaching activities. Even if it was not planned to consider the information in the analysis, we hoped that it would help to further understand the relationship between teachers’ motivational orientations and students’ performance. Unfortunately, this method reveals problems. No direct observation was made. Although we formulated a clear structure beforehand, providing grids the teachers only had to complete, the information the teachers provided were of such inconsistent quality that we were not able to compare and analyse them [74]. Accordingly, we did not consider the grids further.

### Implications

The results of our study reveal research desiderata.

We found, that teachers’ subject-specific enthusiasm is related to students’ performance. To understand how subject-specific enthusiasm culminates in increased students’ performance, it is important to further understand and examine mediating factors. There is evidence that cognitive autonomy support–a characteristic of instructional quality–mediates the relationship between subject-specific enthusiasm and students’ performance [13]. Förtsch and colleagues [87] also found a positive relationship between cognitive activation as an indicator for instructional quality and students’ performance. However, there are also other possible mediators, for instance students’ on-task behaviour and students’ enthusiasm [40].

We know from Gross and colleagues [41–43] that the experience of emotions is related to a certain behaviour (i.e. the expression of emotions). Available research often focuses on either the experienced enthusiasm (e.g., [13]) or the expressed enthusiasm (e.g., [32]). It would be fruitful to simultaneously consider both “levels” when investigating the relationship between teacher enthusiasm and students’ performance. This would be possible in a classroom context and in an experimental design. Previous to the respective lesson or intervention the experienced enthusiasm (e.g., for the respective subject and teaching this subject) should be measured using a questionnaire. Within a classroom context lesson observation should also take part (as the teachers are not trained to show a certain behaviour). Two further aspects are interesting to consider both in classroom contexts as well as in experimental designs. First, Gross and Colleagues [41–43] stated that the tendency to express emotions serves as mediator between the experience of emotions and the expression of emotions. This tendency could also be assessed previous to the lesson/the intervention. Second, it would be fruitful to also consider the students’ perspective (i.e. the perceived teacher enthusiasm). This procedure could provide clarity, if the behaviours, which are considered as enthusiastic (e.g., movements), are indeed perceived as enthusiastic by students.

## Conclusions

Motivation is important for choosing a profession, to remain in the profession as well as to succeed in the profession [88]. Our findings support the assumption that successful teaching requires more than professional knowledge, by revealing a positive relationship between teacher enthusiasm and students’ performance. The strength of this study is that we considered both cognitive and affective domains of teachers’ motivational orientations by examining relationships between students’ performance and both, teachers’ self-efficacy and teachers’ enthusiasm, by using doubly-latent models. To our knowledge, this is the first use of the approach in this field of research. Moreover, we gathered deep insights into the links between teacher enthusiasm and students’ performance by distinguishing between subject-specific enthusiasm and enthusiasm for teaching the subject. The results of our study suggest that teacher education should focus not only on the acquisition of knowledge, but also on the improvement of motivational orientations. This is an important future task for teacher educators, because hitherto, at least in the German system [89], specific support is lacking (especially in the first phase of teacher education).

## Supporting information

S1 Measures Measures applied in the teacher and student sample. (DOCX)

S1 Dataset SPSS-datasets for the teacher and student sample. (ZIP)
