# Supplementary material for: Does motivation matter? – The relationship between teachers’ self-efficacy and enthusiasm and students’ performance
Source: PLoS One. 2018 Nov 21;13(11):e0207252. doi: 10.1371/journal.pone.0207252 (PMC6248951; doi:10.1371/journal.pone.0207252)
Supplement: S1 Measures — (DOCX) [file pone.0207252.s001.docx]

**Measures**

*Please note:* Data are available as supplementary material. The variable names applied are indicated in bold in the beginning of each item.

**Teacher level**

**Self-efficacy** [61]

(4 = fully applies; 3 = largely applies; 2 = does rather not apply; 1 = does not apply at all)

**SW1**: I am convinced that I am able to successfully teach all relevant subject content to even the most difficult students.

**SW2**: I know that I can maintain a positive relationship with parents even when tensions arise.

**SW3**: When I try really hard, I am able to reach even the most difficult students.

**SW4**: I am convinced that, as time goes by, I will continue to become more and more capable of helping to address my students’ needs.

**SW5**: Even if I get disrupted while teaching, I am confident that I can maintain my composure and continue to teach well.

**SW6**: I am confident in my ability to be responsive to my students’ needs even if I am having a bad day.

**SW7**: If I try hard enough, I know that I can exert a positive influence on both the personal and academic development of my students.

**SW8**: I am convinced that I can develop creative ways to cope with system constraints (such as budget cuts and other administrative problems) and continue to teach well.

**SW9**: I know that I can motivate my students to participate in innovative projects.

**SW10**: I know that I can carry out innovative projects even when I am opposed by skeptical colleagues.

**Subject-specific enthusiasm** [62]

(4 = fully applies; 3 = largely applies; 2 = does rather not apply; 1 = does not apply at all)

**EN3**: I am enthusiastic about the subject biology.

**EN1**: I think biology is an exciting subject.

**EN2**: I always try to get students enthusiastic about the subject biology.

**Enthusiasm for teaching the subject** [62]

(4 = fully applies; 3 = largely applies; 2 = does rather not apply; 1 = does not apply at all)

**EN5**: I teach biology with great enthusiasm.

**EN4**: Teaching biology is fun.

**Student level**

*Please note:* The same items were used in pre- and posttest. Variable names ending with _VT refer to the pretest, variable names ending with _NT refer to the posttest.

**Concept maps** [71]

Variable names: **CW3_VT / CW3_NT**

*Set of Given Concepts:*

blue mussel; byssus; egg; eider duck; human; larvae; mussel breed; oyster; sea star; young mussel; worm

*Set of Linking Words:*

becomes; develops; eats; protects; displaces; breeds; sticks together; lives in; builds; warms; pulls

**Questionnaire** [71]

*Items for Procedural System Thinking Assessment (procedural system thinking)*

**VT_M6 / NT_M6**: A North Sea exposition plans to put algae in a ‘blue mussel aquarium.’ Are the blue mussels limited by those algae?

**VT_M9 / NT_M9**: Natural breeds are totally overfished now. How do fishers get their mussels?

**VT_M10 / NT_M10**: Blue mussels and a lot of plankton is arranged in an aquarium. When Sea stars are added,

***□*** *the amount of plankton decreases.****□*** *the amount of plankton increases.****□*** *the amount of plankton remains constant.****□*** *the amount of Blue mussel decreases.*

**VT_M13 / NT_M13**: Oysters are much stronger than Blue Mussels. Imagine Oysters displacing the mussels in a few years. What are the consequences for eider ducks?

**VT_M16 / NT_M16**: Sometimes water stays during low tide because of a hard storm. What are the consequences for Blue Mussels?
***□*** *They have more time to feed and to breathe.****□*** *Sea Stars have more time to feed the mussels.****□*** *They won’t get enough air to breathe.****□*** *They won’t get enough water.*

**VT_M17 / NT_M17**: Are Blue mussels able to survive without water?

***□*** *Yes, no problem, this would work endlessly.****□*** *No, they are not able to survive without the fresh water flow.****□*** *No, they die immediately in the air.****□*** *Yes, but only a few hours.*

**VT_M18 / NT_M18**: Oysters don’t feed on mussels and don’t kill them. Why are they still so threat-generating for mussels?

**VT_M19 / NT_M19**: Why are Blue Mussel breeds important for the Wadden Sea?
***□*** *They offer protection for other animals.****□*** *It’s confusing for predator to pick a single mussel.****□*** *They protect the mussels.****□*** *They offer ground for younger mussels to stick on.*

*Items for Structural System Thinking Assessment (structural system thinking)*

**VT_M1 / NT_M1**: How many Blue mussels can live on a mussel bed?

***□*** *10-100****□*** *100-200****□*** *200-800****□*** *many thousands*

**VT_M2 / NT_M2**: You’re asked to explain how blue mussels feed. Which of those terms do you need? Circle!

‘inlet opening’ air plankton water filtering

chewing ‘outlet opening’ oxygen

**VT_M3 / NT_M3**: Which of those circles is the right one?


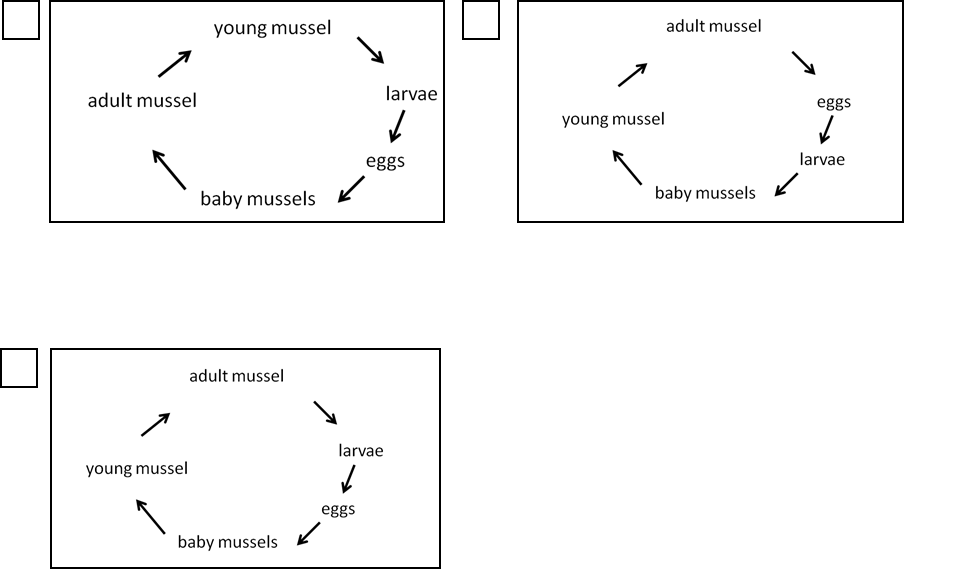


**VT_M4 / NT_M4**: In spring each female Blue mussel produces millions of eggs. Why is such a big number of eggs reasonable?

***□*** *Many eggs are fed therefore they must produce such a big number.****□*** *There is no particular reason for it.****□*** *The mussels feel threatened by predators, therefore they produce such a big number.****□*** *Many eggs are flushed away, therefore they must produce such a big number.*

**VT_M5 / NT_M5**: Why do blue mussels filtrate the sea water?
***□*** *to clean their bodies****□*** *to breathe****□*** *to filtrate food****□*** *to move*

**VT_M7 / NT_M7**: Circle those terms concerning the breathing of blue mussels!

Oxygen Water Oystercatcher Sand ‘outlet opening’ plankton ‘inlet opening’ blue mussel air

**VT_M8 / NT_M8**: Are Blue mussels able to breathe?

***□*** *No, they live underwater.****□*** *Yes, but only at low tide.****□*** *Yes, they make use of the oxygen in the water.****□*** *No, they don’t need to.*

**VT_M11 / NT_M11**: Who eats whom?


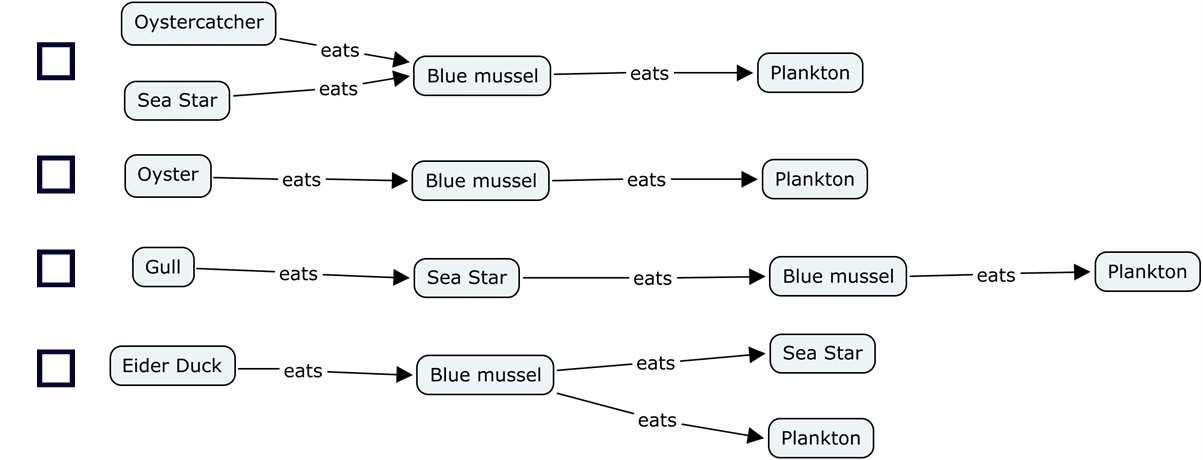


**VT_M12 / NT_M12**: What would happen if the Blue mussels wouldn’t have a foot?

***□*** *They couldn’t escape from the seagulls.*

***□*** *They couldn’t hold on to the ground.*

***□*** *They couldn’t reproduce.*

***□*** *They would slowly sink into the mud.*

**VT_M14 / NT_M14**: How do Blue mussels breed?

richtig

richtig

***□*** *Little mussels grow out of adult mussels****□*** *Larvae grow up inside adult mussels and became baby mussels later on.****□*** *They produce eggs and flush them into the water where they became larvae. Those develop into mussels later on.****□*** *They produce larvae; those become eggs who later develop into mussels.*

**VT_M15 / NT_M15**: Which of these terms are irrelevant for a Blue Mussel’s life? Strike them out!

Sea Star foot sand human air water oxygen shell

**VT_M20 / NT_M20**: How do mussel beds develop?

***□*** *Young mussels are flushed together by the water and stay together as a heap.****□*** *The mussels stick together and thus form big mussel beds.****□*** *Seagulls chase the mussels together thus forming big mussel beds.****□*** *Fishing boats chase the mussels together thus forming big mussel beds.*

**VT_M21 / NT_M21**: What happens to the blue mussels at low tide?
***□*** *They are flushed away with the water; otherwise they would dry up.****□*** *They stay at the bottom the whole tide long.****□*** *They dig themselves into the bottom.****□*** *With closed shells, they survive with a small amount of water.*

**VT_M22 / NT_M22**: What do mussels feed on?
***□*** *Basically, they eat worms and crayfish.****□*** *They filter little particles.****□*** *Plankton.****□*** *Little fishes.*

**VT_M23 / NT_M23**: Circle the predators of Blue Mussels!

Common Seal Flatfish Common Shore Crab Plaice Gull Human Oyster Oystercatcher Shark

**VT_M24 / NT_M24**: How are Blue Mussels able to stick to each other?

*□ with their excrement
□ with salt crystals
□ with byssus
□ with food*

**VT_M25 / NT_M25**: What is the difference between natural mussel beds and mussel breeding farms?

***□*** *Sea stars are removed from mussel breeding farms.****□*** *Natural mussel beds can get very old.****□*** *In mussel breeding farms the mussels are frequently harvested.****□*** *Sea stars are removed from natural mussel beds.*

**VT_M26 / NT_M26**: How do Blue Mussels protect themselves from heat at low tide?

***□*** *They open their shells and cool down through an air draft.****□*** *They back out into the water.****□*** *They dig themselves into the bottom.****□*** *They consume just a little amount of water and rest in a kind of sleeping mode.*
